# Supplementary material for: A decision support system for primary headache developed through machine learning
Source: PeerJ. 2022 Jan 11;10:e12743. doi: 10.7717/peerj.12743 (PMC8759354; doi:10.7717/peerj.12743)
Supplement: Supplemental Information 2 [file peerj-10-12743-s002.docx]

您好! 本问卷是常规的术后随访及临床调研项目，旨在更好的了解您的头痛情况，方便定期随访，及为临床统计提供数据。您的答案无所谓对错。 本问卷填答的资料绝对保密，所以请您根据自己的真实情况填答，非常感谢您的合作!

1. 下面是一些基本信息及一般身体健康状况的问题

编号____________ 姓名 ______________

1 您的年龄 ___________岁
2您的性别 □男 □女
3 您的工作 □农民 □工人 □职员 □学生 □无业 □其他
4 您的身高_________cm

5您的体重________kg

二、下面您将看到有关头痛特征的相关信息，我们想要更多的了解您的头痛特征。

6您头痛病程多久了？ □月 □年

7您头痛发作的平均频率？ □几天一次 □几周一次 □几月一次 □ 几年一次

8每次头痛持续时间是多久？ □数分钟 □数小时 □数天

9头痛的性质是什么样的？（可多选）

□搏动样 □刀割样 □胀痛

□钝痛□分裂样□其他

10头痛的范围是？（可多选）

□一侧□双侧□左右交替□前后交替□全头

11头痛的主要位置在哪？

□前额□后枕部□颞部□眼眶部

12头痛的严重程度？

□轻 □中 □重

13头痛发作之前有没有以下征兆？（可多选）

□没有征兆 □眼前有闪光的东西或者暗点 □部分视野有缺陷

□单侧的手，脚或者脸有感觉异常 □不能言语或者听不懂别人说话

14头痛发作时有没有以下伴随症状？（可多选）

□无症状 □头痛侧眼充血/流泪/鼻塞/流涕

□恶心呕吐 □畏光畏声

□其他

15头痛是否合并以下表现？（可多选）

□平衡障碍，步态和姿势异常 □耳鸣眩晕，听力减退

□腹泻，食欲减退 □抽搐或者突然地意识丧失跌倒

□颈部疼痛 □其他

16活动后头痛有什么变化？

□加重 □不变 □缓解

17头痛的诱因有哪些？（可多选）

□无诱因

□睡眠不足

□睡眠过多

□经期或者月经前后

□饮酒或者进食甜食

□精神紧张

□咳嗽，用力等动作

□外界冷刺激

□性活动

18哪种方式可以使头痛缓解？

□无缓解

□休息睡眠

□服用药物

□其他

19在过去的3个月，头痛天数？

□15天以下

□15-30天

□30-60天

□60-90天

Hello!

This questionnaire is a routine clinical investigation project, aiming to better understand your condition, facilitate regular follow-up, and provide data for clinical statistics. There is no right or wrong answer. The materials filled in this questionnaire are strictly confidential, so please fill in the answers according to your real situation. Thank you very much for your cooperation!

Firstly, Here are some basic information and questions.

Number: _____________ Name______________

1. Ages ___________years

2.Sex ○ Male ○ Female

3. Job ○ Farmer ○ Worker ○ Staff ○ Students ○ Unemployed ○ Others

4. Height (m) ________________________

5.Weight (kg) ________________________

Secondly, you will see some information about headache symptoms, we want to know about the characteristics of your headache.

6. The course of your headache ○Months ○ Years

7. The average frequency of your headaches

○ Once in a few days ○ Once in a few weeks ○Once in a few months ○ Once in a few years

8.The duration when each headache attack ○Minutes ○Hours ○Days

9. The nature of your headache (Multiple choice)

□Throbbing pain□ Stabbing pain □Compression pain

□Dull pain □ Schizophrenia headache □Others

10. The location of your headache (Multiple choice)

○ One side ○ Both sides ○ Alternate left and right ○ Alternate front and back ○ Full head

11. The main part of the headache

□ Frontal □ Occiput □Tempus □ Orbit

12. The severe intensity of your headache ○ Light ○ Medium ○ Heavy

13. Before the headache occurred, did you have the following symptoms? (Multiple choice)

□ No symptoms □spark in front of your eyes □A short period of visual disturbance

□ Paresthesia on one side of the hands, feet or face □Difficulty speaking

14. Is the headache accompanied by the following symptoms? (Multiple choice)

□No symptoms

□Autonomic symptoms such as eye watering, nasal congestion and swelling around the eye

□ Nausea/vomiting

□ Photophobia/phonophobia

□ Others

15. Will your headaches be accompanied by the following symptoms? (Multiple choice)

□ Balance disorders, abnormal gait and posture

□ Vertigo, ringing in ears

□ Diarrhea, loss of appetite

□ Epileptic seizure

□ Neck pain

□ Others

16. The headache changes after activities (such as going up and down stairs, shaking your head or walking)

○ Aggravate ○ Unchanged ○ Relieve

17. The triggers for your headache (multiple choices available)

□ None

□ Lack of sleep

□ Too much sleep

□ Related to the menstrual cycle

□ Drinking alcohol or eating sweets

□ Mental stress

□ Coughing, exerting force, etc.

□ External cold stimulation

□ Sexual activities

18.The alleviative way when headache attacks

○Persistence

○Rest

○Drug

○Else

19. In the past 3 months, the headache days

○ Less than 15 days

○ 15-30 days

○ 30-60 days

○ 60-90 days
